# Taste aversion training can educate free-ranging crocodiles against toxic invaders

**DOI:** 10.1098/rspb.2023.2507

**Published:** 2024-08-14

**Authors:** Georgia Ward-Fear, Miles Bruny, the Bunuba Rangers, Clare Forward, Ian Cooksey, Richard Shine

**Affiliations:** ^1^ School of Natural Sciences, Macquarie University, Sydney, New South Wales 2109, Australia; ^2^ Department of Biodiversity, Conservation and Attractions, Wanneroo, Western Australia 6065, Australia; ^3^ Bunuba Dawangarri Aboriginal Corporation, Fitzroy Crossing, Western Australia 6765, Australia

**Keywords:** *Bufo marinus*, cognition, learning, lithium chloride

## Abstract

Apex predators play critical ecological roles, making their conservation a high priority. In tropical Australia, some populations of freshwater crocodiles (*Crocodylus johnstoni*) have plummeted by greater than 70% due to lethal ingestion of toxic invasive cane toads (*Rhinella marina*). Laboratory-based research has identified conditioned taste aversion (CTA) as a way to discourage consumption of toads. To translate those ideas into landscape-scale management, we deployed 2395 baits (toad carcasses with toxin removed and containing a nausea-inducing chemical) across four gorge systems in north-western Australia and monitored bait uptake with remote cameras. Crocodile abundance was quantified with surveys. Free-ranging crocodiles rapidly learned to avoid toad baits but continued to consume control (chicken) baits. Toad invasion at our sites was followed by high rates of crocodile mortality (especially for small individuals) at a control site but not at nearby treatment sites. In areas with high connectivity to other waterbodies, repeated baiting over successive years had continuing positive impacts on crocodile survival. In summary, we succeeded in buffering the often-catastrophic impact of invasive cane toads on apex predators.

## 1. Introduction

Although large-bodied apex predators typically exhibit low population densities, such species can strongly affect biological communities. For example, loss of apex predators can increase the abundance of prey and change the intensity as well as the spatial distribution of areas subject to intense herbivory (e.g. wolves and elk in Yellowstone National Park [1]). Tragically, up to 75% of large carnivore species are declining worldwide, causing complex and sometimes devastating impacts on the ecosystems in which they are embedded [2,3]. Recognition of the important ecological role of apex predators has stimulated intensive efforts to reverse such declines by mitigating humanwildlife conflict (e.g. predation by felids, canids and bears on domestic livestock [4]) and/or by establishing protected areas where agricultural activities are prohibited [5].

The challenge of maintaining large predators is even greater when these animals are imperiled by invasive species rather than by humans because reserves and elimination of hunting cannot solve the problem. In some cases, direct culling of invaders may lessen the impact on wildlife—but many invasive species are impossible to control [6]. Thus, for example, the fungus responsible for exterminating many anuran species is now found almost worldwide, with no feasible options to restrict its distribution [7]. Likewise, the spread of toxic cane toads (*Rhinella marina*) through tropical Australia has continued unabated despite control efforts, causing local extirpation of apex predators [8,9]. In such systems, where the threatening process cannot be controlled, the only option for the conservation of apex predators is to facilitate their coexistence with the threat.

Conditioned taste aversion (CTA) offers great promise in this respect. The method relies upon the widespread ability of animals to learn to avoid food whose consumption induces nausea [10]. Conservation programmes are beginning to capitalize on this innate behavioural mechanism to ‘train’ an array of taxa against consuming a target item (e.g. a novel prey species). Strategies often involve adding nausea-inducing chemicals to render food items unpalatable and aim to address issues ranging from species management (e.g. deterring crows from consuming eggs of endangered birds [11]), management of nuisance behaviours (e.g. deterring black bears from pilfering army rations: [12] and reducing direct human–wildlife conflict (depredation of agricultural crops: [13], or stock). For example, we might add nausea-inducing chemicals to the carcasses of livestock killed by lions or wolves, to teach the predators that such prey is best avoided and thus reduce the frequency of ‘revenge killing’ by pastoralists [14,15]. Similarly, predators at risk of death from ingesting highly toxic invasive prey can be taught that such novel prey items should not be consumed in the future [10]. In some cases, a single nausea-inducing meal can induce a predator to avoid that type of prey for many months [16,17].

CTA has been demonstrated in several (but not all) apex predators whose populations have been reduced by the invasion of cane toads in Australia. Laboratory studies and small-scale field trials (based on following the fates of radio-tracked predators) have provided proof-of-principle that carnivorous marsupials (e.g. quolls, planigales) and reptiles (e.g. varanid lizards, scincid lizards and crocodiles) can learn to recognize and avoid toads as prey [16–20]. Nonetheless, upscaling those results to landscape-level deployment of CTA-inducing stimuli is a major logistical challenge in the remote and rugged landscapes of north-western Australia.

In the current paper, we describe a study designed to buffer the impact of toad invasion on freshwater crocodiles. In a recent complementary study, we described successful CTA training of free-ranging varanid lizards by a consortium of researchers, wildlife managers and indigenous custodians [21]. Despite their similar aims, the two studies involved different predator species (lizards versus crocodiles), in different habitats (savannah woodland versus waterbodies) and using different stimuli (small live toads versus parts of the carcasses of adult toads). In combination, the two studies demonstrate not only the value of CTA, but also the need to take into account the vulnerable species' habitat and behaviour when developing methods to deliver aversion-inducing stimuli.

## 2. Methods

### (a) Study species

The Kimberley region, in the monsoonal wet-dry tropics of north-western Australia, comprises a mosaic of savannah woodland, rocky escarpments and gorges with open riparian zones dominated by melaleuca and acacia overstories around large, seasonally flowing river systems. Up to 95% of annual rainfall occurs in the wet season (November to April; long-term average 798 mm) rather than the dry season (May to October: long-term average 35 mm [22]). As a result, many river systems dry up to a series of more-or-less-isolated pools during the middle part of the year.

**Figure 1.**
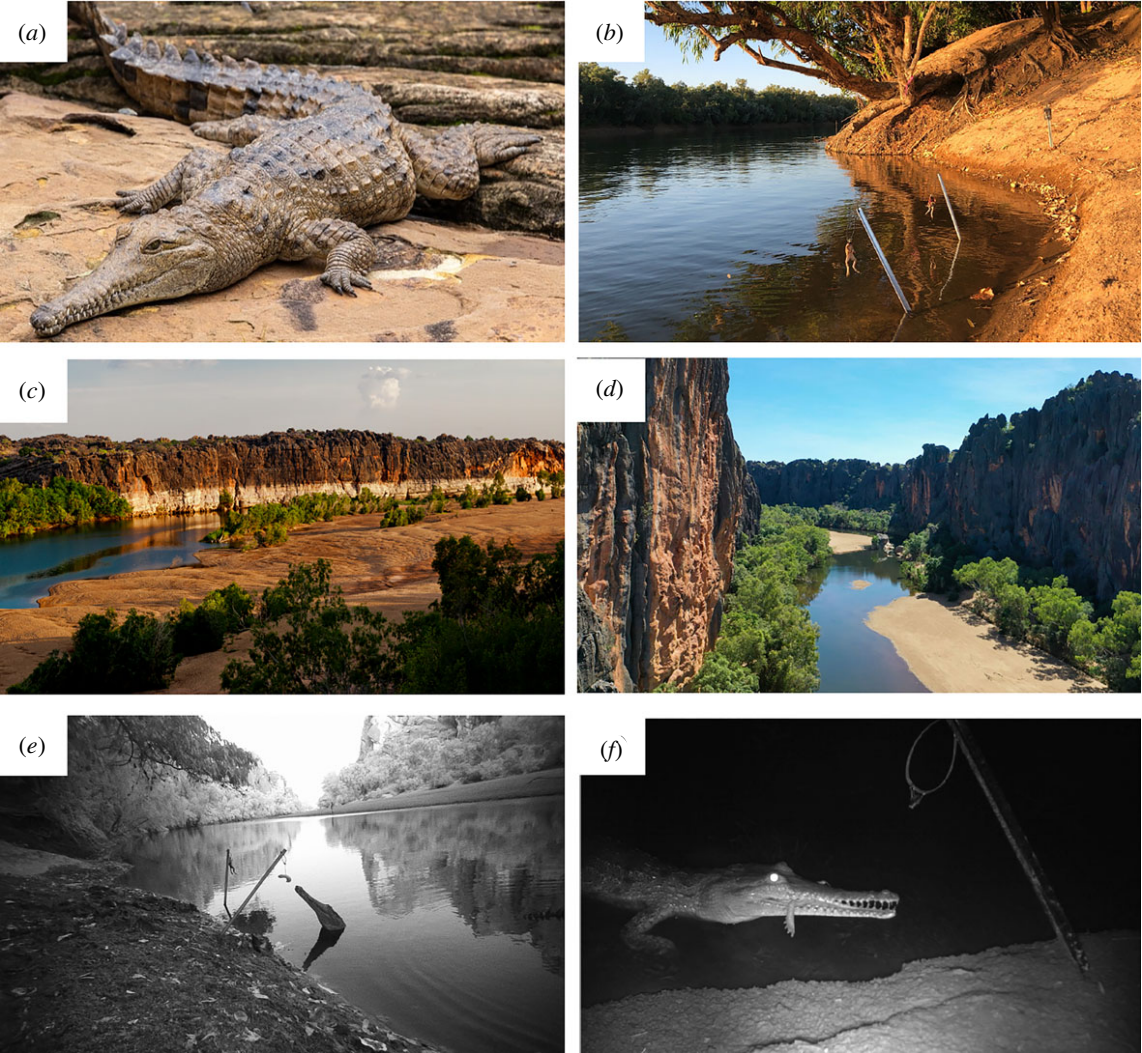
(*a*) Freshwater crocodiles (*Crocodylus johnstoni*) are large predators in freshwater systems across tropical Australia. (*b*) The taste aversion baiting apparatus set up and baited at Scrootens waterholes, with a remote camera to detect consumers. (*c*) Dangguu Geikie Gorge National Park in the central Kimberley, where cane toads arrived in 2019. (*d*) Bandilngan (Windjana Gorge) National Park, where toads arrived in 2021. (*e*) A crocodile investigates freshly set baits (control and treatment) on dusk, caught on remote camera. (*f*) A crocodile consumes a cane toad bait laced with lithium chloride, caught on remote camera. Photo credits: (*a*) Ken Griffiths; (*c*) Ripple100; (*d*) Air Kimberley; (*b*,*e*,*f*) Miles Bruny (Department of Biodiversity Conservation and Attractions).

The freshwater crocodile (*Crocodylus johnstoni*) is the largest resident freshwater predator in northern Australia [23] (figure 1*a*). Males can attain 3 m in total length and weigh up to 100 kg; females grow to 2 m in total length and up to 40 kg [24]. These crocodiles mostly take aquatic prey such as prawns, fish, amphibians and reptiles, but also forage terrestrially [24,25]. Cane toads (*Rhinella marina*; formerly *Bufo marinus*) are large bufonids that were introduced into Australia as a biocontrol agent in 1935 [26]. They failed at their original purpose [27], but have spread throughout tropical Australia. The toads are highly toxic and exude powerful cardiotoxins from the parotid glands when threatened [26]. Most Australian predators lack evolutionary history with bufonids (and their associated bufotoxins), and die within minutes of trying to consume a cane toad [8]. As a result, populations of medium to large anurophagous predators have been decimated in the wake of the cane toad invasion [8,9]. Many populations of freshwater crocodiles have exhibited severe declines following the arrival of cane toads [28–31], whereas other populations (especially those in which terrestrial foraging is rare) have been unaffected (in the short term at least [25,32,33]). Freshwater crocodiles are culturally important to First Nations communities across tropical Australia, including the Bunuba people of central Kimberley.

### (b) Study sites

**Figure 2.**
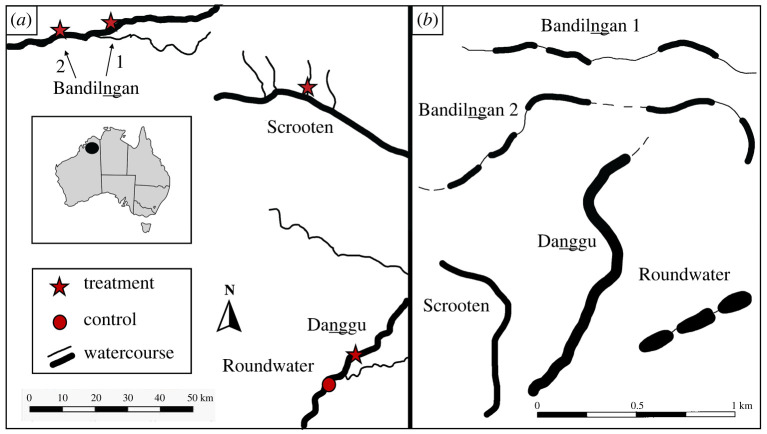
(*a*) Scale map of the relative locations of treatment and control sites across the Fitzroy Valley Region: Bandilngan 1, Bandilngan 2, Scrooten, Danggu and Roundwater. (*b*) The orientation, lengths and configuration of pools at each site during baiting trials in September 2021. Dotted lines represent continuing or inaccessible sections of gorge. All sites are to scale, except Danggu, which is double the size depicted here.

We worked with crocodile populations throughout the Fitzroy Valley region in the central Kimberley, across Bunuba Country, as the cane toad invasion moved through that area in 2019–2021. Our treatment zones were in Bandilngan (Windjana Gorge) National Park (17°24′2″S, 124°56′4″E) along the Lennard River (two large study sites: ‘Bandilngan 1 and 2’), in Danggu Geikie Gorge National Park (18°04′38″S, 125°42′49″E) along the Fitzroy River (one large study site: ‘Danggu’; figure 1*c*) and at Scrootens waterholes (17°32′33″S, 125°32′08″E; one large study site: ‘Scrooten’). Each ‘site’ comprised sections of river stretching through gorge systems, which may have contained multiple pools and were fringed by melaleuca woodland (figure 2). Near Danggu we recognized an additional site: an isolated series of pools that we used as a control area (Roundwater), 5 km downstream of the Danggu treatment area. We measured pool sizes and site areas (as they were during the month of baiting treatments) as polygons from Google Earth, to calculate population densities of crocodiles and design the baiting strategy in real time.

All of these areas are continuous (connected) river systems during the monsoonal period but during the dry season, they shrink to small pools that may contain hundreds of crocodiles. However, crocodiles can move between adjacent pools during the dry season by traversing intervening areas along the dry riverbed, such that a series of nearby pools within a site may effectively constitute one large ‘waterbody’. We classed local populations as either ‘closed’ (a discrete isolated ‘waterbody’ with no emigration or immigration) or ‘open’ (treatment site part of a long continuous section of river; or adjacent pools inaccessible to us such that crocodiles could move in or out of the range of surveys).

### (c) Experimental design

The most powerful design (equal numbers of before-after-control-impact sites) was not achievable in our study, because: (1) the toad invasion had already reached some areas and we had ‘before toad’ data on crocodile abundance for only a single area (see below); and (2) the near-certainty of high mortality based on observations in similar systems across tropical Australia [28,29,31], and in Danggu Geikie Gorge National Park by the time our work began, forced us to implement treatment in as many sites as possible, on ethical grounds. In planning with management authorities and indigenous traditional owners, we agreed that one ‘control’ (unbaited) site could be used also (‘Roundwater’; see below).

### (d) Abundance of crocodiles

From July to November of 2021, we ran monthly nocturnal spotlighting surveys of crocodiles [34,35] from the riverbanks at all sites, to estimate population sizes. During the spotlighting survey, multiple counts of ‘eye shine’ were conducted around each pool, from various vantage points, and the highest count was accepted. Surveys moved upriver each night and were repeated two or three times a week each month. We also counted any cane toads present. We averaged the last three spotlight counts prior to November (when hatchling crocodiles emerge) as estimates of local population densities for our statistical analyses. Populations generally remained static across the week of surveys and between months after July/August when pools are discrete and areas are well-defined (and easy to spotlight). Within each site, we worked at multiple pools or sections of the river, and each was assessed independently. We then combined adjacent pools into larger waterbodies or ‘sites’ and calculated their total densities of crocodiles (the number of crocodiles per water surface area as measured from polygons).

### (e) Assessing crocodile mortality due to cane toad ingestion

Any crocodiles found dead or dying were examined. Our data are most extensive for Danggu Geikie Gorge National Park, where rangers take visitors on tours in a 10 m boat, 5 km up the gorge and then back again, three times a day between May and October. The rangers began collecting data on crocodile deaths in 2018, shortly before cane toads arrived in the area. Rangers also engaged with tour groups daily in Bandilngan (Windjana Gorge) National Park. These supervised tours included walks up and down the gorge (in both sites 1 and 2) throughout the day from May to October during 2021 and 2022. Members of the public were asked to report any crocodile carcasses to rangers. Given the high flow of ‘tourist traffic’ through the gorge, most crocodile carcasses would have been detected and reported. We also conducted surveys for carcasses at Scrooten and Roundwater multiple times each month through the late dry season in 2020, 2021 and 2022.

Wherever possible, crocodile carcasses were collected, morphometrics recorded (mass; snout-to-vent length, SVL) and a necropsy was performed to ascertain the cause of death. If this was not possible, the animal's SVL was estimated to the nearest 200 mm. We scored death as due to ingestion of a cane toad if there was a toad in the stomach, or if the predator was uninjured, in good condition and showing no signs of ill health [17,29]. Crocodiles with toads in their stomachs also often exhibited large haemorrhages within the cardiovascular and digestive systems. Toad-poisoned crocodiles sometimes were found while still alive, floating on the water surface and exhibiting muscular seizures, continuous ‘death rolls’ underwater, bouts of extreme lethargy, limb paralysis, impaired coordination, and unresponsiveness with gaping mouth. We were unable to save any of these animals.

### (f) Eliciting taste aversion

Baiting trials were conducted all sites in September and October of 2021. We ran a repeat baiting at Bandilngan sites 1 and 2 in September 2022 (but not other sites), to explore the longevity of our intervention in a large population experiencing toads for the first time. We also wanted to clarify population dynamics between years at dry-season waterbodies in this important gorge ecosystem, which would allow us to design future conservation protocols. Trials were timed to coincide with late dry-season peak overlap between crocodiles and toads when pools were relatively isolated. Based on our spotlight counts just prior to trials, we calculated the number of bait stations required in each area to keep the ratio of crocodiles to bait stations below 2 : 1, while maintaining 50–100 m distance between stations along both sides of the waterbodies. Our rationale for these calculations was twofold and based on the spatial ecology detected during spotlight surveys. Pragmatically, we aimed to maximize the number of bait stations available to crocodiles while limiting competition between animals, to ensure wide application of the baiting strategy. Ethically, we aimed to minimize the chance of single animals consuming too many baits and becoming overly ill.

At each bait station, two 90 cm metal stakes were placed 2 m apart, angled outwards at 30°, with their bases in water to prevent ant attack (following methods in Aiyer *et al*. [35]). Baits swung freely 100 mm above the surface of the water to be visible to crocodiles while discouraging consumption by freshwater prawns or fish. Baits were suspended by bulldog clips, allowing easy release when pulled (figure 1*b*).

To induce generalized aversion to cane toads, each bait consisted of the fresh carcass of an adult toad (approx. 100 g, 70 mm) with internal organs, head and parotid glands removed to render it non-lethal [36]. To ensure the baits would elicit taste aversion, we added 5 ml of lithium chloride (LiCl; concentration 0.5 Mol [37]) via intramuscular injection into both back legs of the cane toad carcass. We honed the dosage rate through pilot studies with captive crocodiles. Generally, chemicals used to elicit taste aversion should be odourless and tasteless, such that the target aversive ‘cue’ (in this case, the scent or taste of cane toads) remains untainted [10]. Because LiCL is salty, it is often (in laboratory studies at least) administered via injection or drenching into the target animal. This was not possible in the context of our *in-situ* conservation study, with large crocodilians. However, crocodiles were unlikely to detect the LiCl in baits, because (a) they are ambush predators, initiating fast attacks from the water then consuming toad-sized prey whole, and (b) they spend time in seasonally brackish environments and are accustomed to consuming prey within saline water [23]. A paired control bait (chicken neck approx. 40 g, 50 mm) was hung on one arm at each station on day 1 and day 5 of trials, so that visiting animals could choose between both bait types. This design allowed us to distinguish between satiation and taste aversion at the end of the trial.

To identify consumers of bait, we set up a remotely triggered wildlife camera (Little Acorn; model Ltl6310WmC) on the riverbank behind every second or third bait station (depending on the number of stations at each site). Cameras were set to high sensitivity and recorded a burst of three photos every time they were triggered by motion or a thermal differential. We later reviewed photographs using IrfanView v4.62 and scored the taxa to species level if possible.

Baits were deployed from canoes for five successive nights, between 1500 and 1700 h, and checked between 0600 and 0800 h the next morning. We recorded whether or not baits were eaten, and then removed all remaining baits and replaced them that afternoon (see Aiyer *et al*. [35] for details).

### (g) Analysis of data

For all data from baiting trials, we ran binomial Generalised Linear Mixed Models with a logit link function, using the GLIMMIX procedure in SAS v9.4 M8 (SAS Institute, Cary, NC). When comparing multiple sites, we incorporated ‘bait station ID’ nested within ‘site ID’ as a random factor in the analyses, to account for pseudo-replication and repeated measures.

To assess whether rates of toad bait consumption declined over the five-day baiting period (demonstrating aversion), our dependent variable was bait ‘Eaten/Remain’ and we ran an analysis including the independent variables of trial day (1–5), crocodile density and population connectivity (open/closed). We included interaction terms that might change patterns of bait offtake from those expected with the development of learned aversion: trial day × crocodile density (increased competition throughout the trial), trial day × population connectivity (immigration of naive individuals during the trial), and crocodile density × population connectivity (to compare general levels of activity and bait offtake).

To compare the offtake of toad versus control chicken baits on day 1 and day 5 of trials (further demonstrating learned aversion), our dependent variable was bait ‘eaten/remain’ and we ran an analysis including the independent variables of trial day (1–5), treatment (toad/chicken), crocodile density and population connectivity (open/closed). We included interaction terms that might influence whether baits were differentially consumed during the trial: trial day × treatment (choice over time: satiation [neither bait consumed on day 5] versus learned avoidance [decreased consumption of toad baits only on day 5]), trial day × treatment × crocodile density (increased competition overriding learning on day 5), and trial day × treatment × population connectivity (immigration of naive individuals during the trial eating equal numbers of baits on day 5). We clarified whether offtake of both bait types declined over time by conducting GLMMs as above, with the independent variable of trial day, analysing chicken and toad datasets separately.

To compare the response of crocodiles in Bandilngan (Windjana Gorge) National Park to baiting trials conducted in 2021 versus 2022, we pooled data together for each year (i.e. sites 1 and 2, because sites were not as discrete in 2022). Our dependent variable was bait ‘eaten/remain’ and we ran a full factorial analysis including the independent variables of trial day (1–5), year (2021/2022) and the interaction between trial day and year. ‘Bait station ID’ was included as a random factor in this analysis.

To investigate the effect of baiting on crocodile deaths in Danggu Geikie Gorge National Park (where we began to witness crocodile mortalities in 2018), we ran a logistic regression analysis with a binomial distribution and logit link function, to model the relative numbers of dead crocodiles (counts of carcasses) compared to live crocodiles (from population spotlighting counts; i.e. the trials/events ratio) in each site (treatment versus control) and time period. The independent variables were treatment (baited versus unbaited control), intervention status (pre-intervention [2018–2020] versus post-intervention [2021–2022]) and the interaction between the two. Analyses were conducted in SAS 9.4 (SAS Institute, Cary NC).

To determine whether the cane toad invasion imposed size-selective mortality, we compared mean SVLs of dead crocodiles to live crocodiles. Data from live animals (*n* = 209) were collected during monitoring programmes at Bandilngan (Windjana Gorge) National Park over four years pre-cane toad invasion (2014–2018). In that catch-and-release programme, the crocodile population was sampled from the lower sites in the gorge extensively each year via seine netting of dry-season pools (selecting a random sample of individuals), with morphological measures taken prior to marking and release. Throughout that period the population was stable and displayed a normal size distribution (Department of Biodiversity Conservation and Attractions, unpublished data, 2023). We compared the means using a one-way ANOVA in JMP v16 (SAS Institute, Cary, NC).

## 3. Results

### (a) Abundance of crocodiles

Bandilngan site 1 (which was deep into the gorge system) had low densities of crocodiles in isolated (closed) populations (i.e. pools were generally separated by greater than 100 m of sandy habitat) whereas Bandilngan site 2 had high densities of crocodiles and was considered an open population because crocodiles could easily move into adjacent pools and lower sections of the river. Danggu had low densities of crocodiles and was an open population because our sites were directly connected to a long stretch of the Fitzroy River. Scrooten had high densities of crocodiles and was an isolated closed population (greater than 2 km from the nearest other waterbody; table 1).

**Table 1.** Information about each ‘site’ at the time of baiting trials in 2021. The local crocodile population, the number of bait stations and total ‘waterbody’ size in square metres (i.e. pool surface areas calculated from Google Earth polygons and combined across each site). Crocodile to bait station ratios were kept below 2 : 1. Relative crocodile densities were calculated per square km, as is common for river surveys, although are inflated due to a large proportion of the population being restricted to smaller dry season pools. We classified treatment sites as ‘high’ or ‘low’ density (relative to each other) and identified whether populations were ‘open’ or ‘closed’ to emigration or immigration across the baiting period. Roundwater was a control site and was only used in analyses on death rates pre- and post-trial.

waterbody ID	number of ‘pools’	combined waterbody size (sqM)	total crocodiles	no. bait stations	relative no. Croc/sqkm	Croc/bait station	relative croc. density	population connectivity
Scrooten	1	10 331	36	25	3485	1.4	high	closed
Danggu	1	696 810	152	90	218	1.7	low	open
Bandilngan 1	3	24 648	14	33	568	0.4	low	closed
Bandilngan 2	5	40 105	102	59	2543	1.7	high	open
Roundwater	3	11 563	22	N/A	1903	N/A		

### (b) Mortality of crocodiles due to ingestion of cane toads

**Figure 3.**
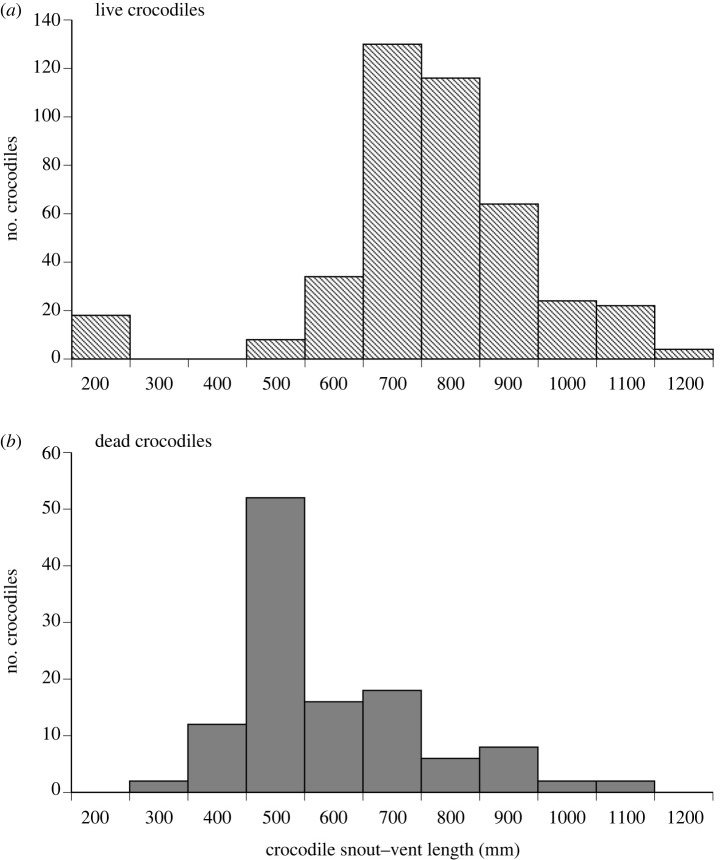
Comparison of the distribution of body sizes (snoutvent length; SVL) in (*a*) live freshwater crocodiles from the Fitzroy Valley and (*b*) crocodiles killed by ingesting cane toads at nearby Danggu Geikie Gorge National Park.

We recorded 187 dead or dying freshwater crocodiles over the course of this study, of which 75 were necropsied. All of the dead crocodiles for which we could confidently assess cause of death either through necropsy or other indicators of unexpected demise (see [14]) had been fatally poisoned by consuming a cane toad (necropsies on *n* = 4 of 4 mortalities in Bandilngan, 4 of 4 in Scrooten, 59 of 157 in Danggu, 8 of 22 in Roundwater). The mean SVL of dead crocodiles was lower than that of the normally distributed wild population of live conspecifics (634 mm, range 322–1120 mm, versus 816 mm, range 211–1160 mm; *F*_1,268_ = 49.57, *p* < 0.0001; figure 3).

### (c) Impact of baiting on feeding behaviour of crocodiles

From our remote-camera images, the only species consuming baits were freshwater crocodiles (figure 1*e,f*). Most images showed single crocodiles, but on at least 12 occasions when densities were high, we saw larger crocodiles aggressively excluding smaller individuals from the vicinity of the bait. We also saw individuals waiting around the bait stations when we rebaited in the afternoons. In one instance, we did not bait four stations for two consecutive days because we felt threatened by crocodiles waiting there.

Consistent with the development of taste aversion, the number of toad baits eaten declined across the five-day baiting period. Combining data for all sites, means and ranges of the proportion of baits taken per day were: day 1 = 92% (range 88–96%), day 2 = 78% (range 65–90%), day 3 = 52% (range 32–87%), day 4 = 50% (range 15–72%), day 5 = 51% (range 20–80%) (*F*_1,1086_ = 26.28, *p* < 0.0001).

**Figure 4.**
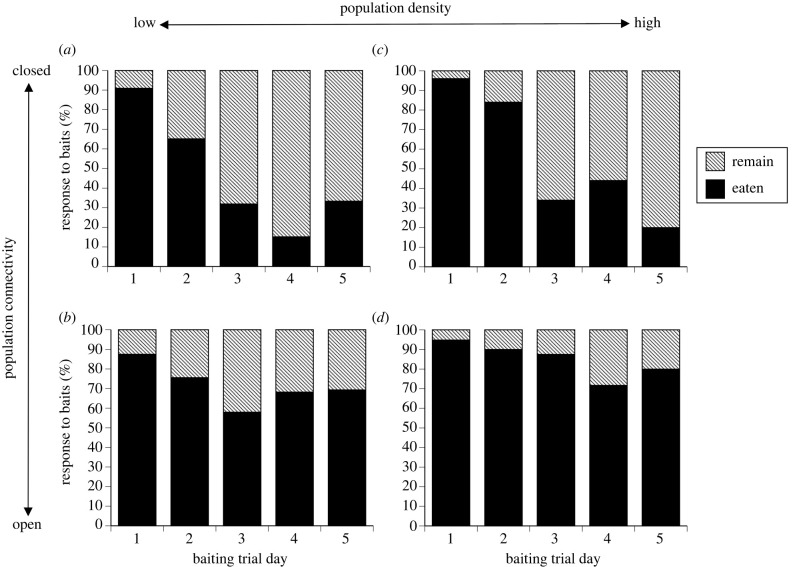
Consumption of taste aversion baits by freshwater crocodiles over 5-day trial periods. The rate of bait consumption varied as a function of crocodile density, and the degree of connectivity to other crocodile populations. Consumption is expressed as the percentage of baits eaten out of the total deployed each day. Trials ran for five consecutive days at each location.

The degree of connectivity of a population to adjacent sites influenced the pattern of bait offtake through time (population connectivity [open versus closed] × trial day: *F*_1,1086_ = 12.17, *p* = 0.0005), but crocodile density did not (density × trial day *p* > 0.1). Closed populations showed rapid declines in rates of bait consumption whereas open populations showed slower declines (figure 4). This effect of connectivity also interacted with crocodile abundance (open/closed × density: *F*_1,1086_ = 6.79, *p* = 0.009) because open populations with high densities (i.e. the situation where total crocodile numbers were highest) showed the highest levels of activity and least decline in bait offtake (figure 4).

**Figure 5.**
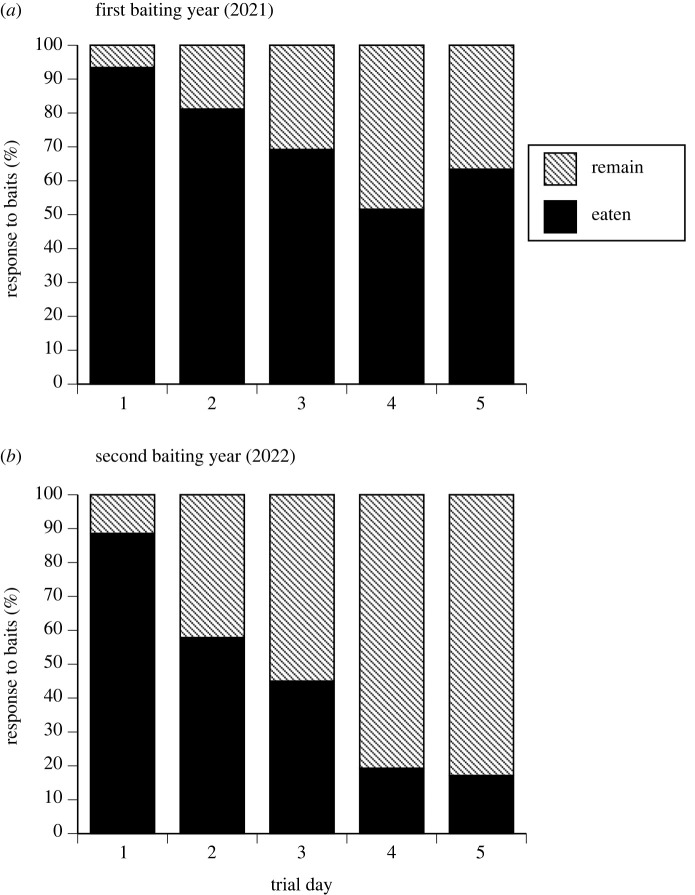
Comparison of crocodile responses to baiting trials conducted in Bandilngan (Windjana Gorge) National Park during (*a*) the first year (2021) and (*b*) the second year (2022) of the trials. Consumption of taste aversion baits by crocodiles is expressed as the percentage eaten of the total deployed each day. Trials ran for five consecutive days, during the late dry-season (September).

Data from Bandilngan (Windjana Gorge) National Park allow us to examine the impacts of baiting at the same sites in successive years (2021 as the toads arrived, and at the same time in 2022). In 2022 the river did not separate into two discrete sites, so for this analysis we pooled all data from sites 1 and 2 into a combined ‘Bandilngan’ site for 2021 and then for 2022. Both years showed declining rates of bait offtake over the five-day trial, but the interaction between trial day and baiting year (*F*_1,1093_ = 14.62, *p* = 0.0001) shows that the decline in bait offtake happened faster and was larger in the second year of baiting (figure 5). Spotlighting counts of crocodiles in Bandilngan were similar in 2021 and 2022 (average of 130 crocodiles seen in the late dry-season).

**Figure 6.**
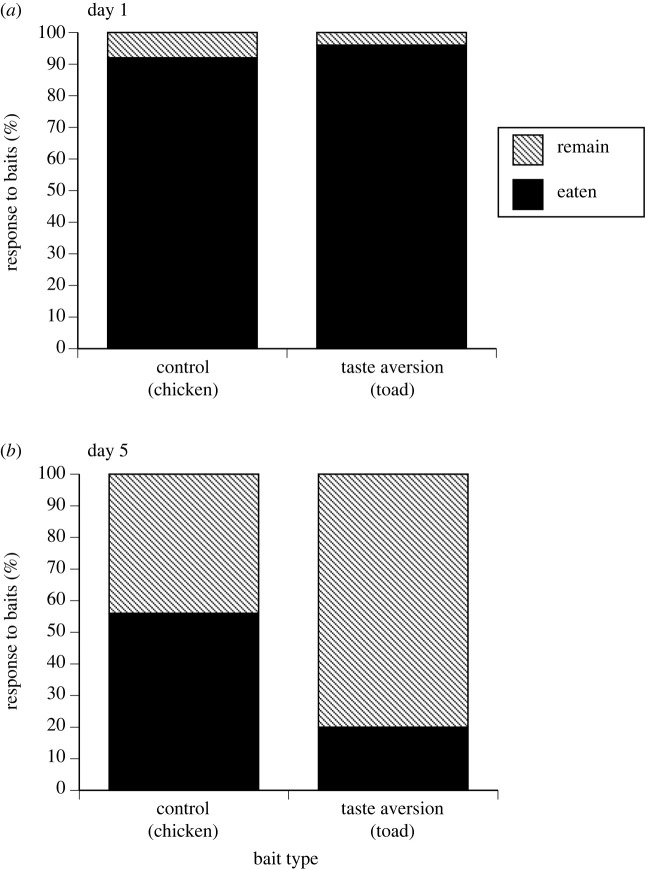
Consumption of control (chicken) and taste aversion (toad) baits by freshwater crocodiles at Scrooten on (*a*) day 1 and (*b*) day 5 of the baiting trial.

Evidence that reduced bait uptake through time was due to taste aversion not satiation comes from the comparison between rates of offtake of toad baits versus control (chicken) baits on day 1 versus day 5 of a trial. Chicken was only provided on the first and last day of the baiting trials. Combining data from all sites, offtake was lower for both bait types on day 5 versus day 1 of those trials (chicken: *F*_1,410_ = 20.15, *p* < 0.001; toad: *F*_1,410_ = 45.18, *p* < 0.001). However, the interaction between trial day (1 versus 5) and bait type (toad versus control/chicken) was the strongest influence on whether or not a bait was eaten (*F*_1,623_ = 4.42, *p* = 0.036). Toad and chicken baits were eaten in equal numbers on day 1 of trials, whereas toad baits were eaten significantly less often than chicken (control) baits on day 5. The strongest response was seen in the high-density closed population where animals consumed 76% of control baits and 26% of toad baits on day 5 (compared with 92% and 96% respectively on day 1; figure 6). However, neither population density nor population connectivity alone influenced the main interaction between trial day and treatment (all *p* > 0.05); in all scenarios, crocodiles were less likely to eat toad baits than control chicken baits on day 5.

### (d) Impact of baiting on rates of crocodile mortality

We describe patterns separately for subsets of our sites, as follows.

#### (i) Bandilngan 1 and 2, and Scrooten

We baited in 2021, the first dry-season after cane toads arrived. We recorded one toad-induced crocodile mortality in Bandilngan 1 and 2, prior to the baiting, and a further three deaths during the trials (all due to ingestion of wild adult toads rather than our baits, confirmed by necropsy). Following baiting, no further crocodile mortalities were detected at Bandilngan (Windjana Gorge) National Park, during either 2021 or 2022. We recorded four dead crocodiles at Scrooten in 2021: three prior to trials, one during trials and all due to ingestion of wild toads, not baits. We recorded no crocodile deaths in 2022.

#### (ii) Danggu and Roundwater

**Figure 7.**
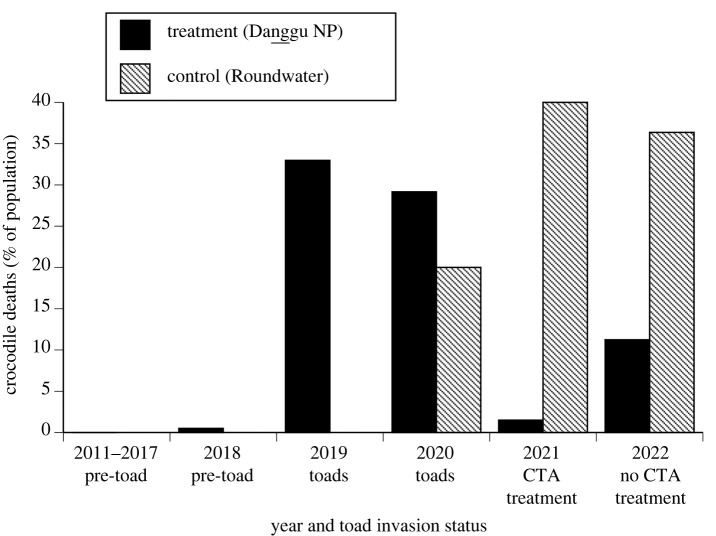
Mortality of freshwater crocodiles due to ingestion of toxic cane toads at a treatment site (Danggu Geikie Gorge National Park) and a control site (Roundwater), before and after deployment of taste-aversion baits (‘CTA treatment’). Mortalities are expressed as the percentage of the local crocodile population, based on spotlighting counts. Raw data on mortality are presented in the Results section.

The most extensive evidence on the impact of baiting was obtained from Danggu Geikie Gorge National Park, where standardized boat surveys have been operating for over a decade. The main frontline of cane toads arrived there in 2019, prior to our baiting. Before cane toads arrived, the Bunuba people had not seen a dead crocodile on the river between 2011 (when standardized boat surveys began) and 2017. One dead crocodile was seen in 2018 (0.5% of the local population estimated from spotlight counts), however, this could have been mortality induced by one of the first invading cane toads. By contrast, rangers found 60 dead crocodiles (33% of the estimated population) in the target section of river in 2019, after toads arrived, and 63 (30% of estimated local population) in the same area in 2020. Crocodiles died in the mid- to late-dry-season from July to November; the onset of deaths was delayed after a wet-season with unusually heavy rainfall. In 2021, the year of our baiting, rangers found 12 dead crocodiles in total, but only three of those died after we concluded our trials (i.e. toad-induced mortality of 1.5% of the population, post-baiting). A year later, in 2022, rangers found 21 dead crocodiles (11% of the estimated local population). At Roundwater (the nearby control population) we found 6 (20%), 8 (40%) and 8 (36%) dead crocodiles in 2020, 2021 and 2022 respectively. Thus, crocodile mortalities immediately declined by 95% post-baiting in our southern treatment area Danggu Geikie Gorge National Park, but increased in a nearby control area (Roundwater), generating an interaction between treatment (baited versus control sites) and time (pre-intervention/post-intervention) (*n* = 8, df = 1, *χ*^2^ = 14.27, *p* < 0.001; figure 7).

## 4. Discussion

In many rivers across tropical Australia, populations of freshwater crocodiles have crashed due to lethal toxic ingestion of invasive cane toads. In some cases, population declines have been substantial (e.g. 77% decline [29]; 65% decline [30]; 70% decline [31]) with toad-induced mortality still occurring at some sites more than a decade after invasion [38]). High mortality rates in our own study system prior to baiting (and in our single unbaited control area) suggest that the Fitzroy River crocodiles were in similarly great peril. By deploying baits, however, we induced conditioned taste aversion in free-ranging crocodiles (as evidenced by reduced rates of toad-bait uptake through time) and buffered the populations against the threat posed by invading toads (as evidenced by low mortality rates of crocodiles post-baiting).

Our experimental design was constrained by logistical factors (difficult terrain and climate), ethical considerations (urgent need to curtail wave of mortality) and socio-political factors (solutions needed to be acceptable to diverse stakeholders). Such challenges are commonly encountered in attempts to translate the results of laboratory research into management in the field, and our results provide an encouraging example of finding a compromise between ideal experimental designs and the reality of wildlife management in a culturally diverse environment. Close collaboration between stakeholders and management agencies provided the local knowledge and capacity needed to produce interventions with conservation benefits for significant crocodile populations as we trialled this methodology. Understanding the population dynamics of target species, as well as local ecology and annual weather cycles, is crucial to designing strategies and planning taste-aversion deployments for maximum impact.

Our observations are broadly consistent with previous studies that have documented toad-induced mortality of crocodiles in other river systems [29–31]. Similarly, other studies have detected size-biased mortality [29,30] but have not explored possible mechanisms for this or discussed their potential evolutionary consequences. Cane toads are so toxic that every encounter with an adult cane toad is likely to be fatal to a freshwater crocodile of any size [27]. Thus, size-selective mortality probably reflects encounter rates. For example: (a) intraspecific aggression may push smaller individuals to the river margins where toads are most common; (b) diet and behaviour shift ontogenetically such that younger individuals are more likely to feed at the river margins, and hence on frogs/cane toads; and/or (c) female crocodiles (smaller in body size than males) spend more time on land between July and October, moving back and forth along river banks while they breed and nest. Regardless of the causal factors, the invasion of cane toads likely exerts strong phenotypic selection within crocodile populations, either via mass mortality events or over the course of slower population declines. Such selection could influence the demography of crocodile populations either by removing younger individuals (that predate anurans more frequently [39]), certain behavioural phenotypes (e.g. bolder, or more exploratory individuals, or those that forage on land rather than in the water [25,30]), or nesting females (which can take 15 years to sexually mature [23]). All these scenarios could impact the trajectory and resilience of crocodile populations to future threats. Few studies have considered how the selective forces imposed by invasive species can alter patterns of local adaptation [40]. This may be especially pertinent in landscapes where systems fluctuate seasonally, annually, and across small spatial scales, such as the rugged Kimberley environment in the wet-dry tropics. An invasion of toxic prey could quickly erode diversity in the behavioural adaptations of naive predator populations (such as local breeding ecology, opportunistic foraging, and exploratory behaviour) that enable generalist predators to thrive in stochastic environments [25,41].

Our findings highlight the importance of investigating the nuanced impacts of invasive species using appropriate experimental designs. For example, the high vulnerability of smaller crocodiles (for various reasons) means that attempts to quantify demographic impacts of toad invasion via before-and-after surveys (if mortality cannot be recorded as it happens) need to monitor the abundance of crocodiles of all age classes and at appropriate times of year. In earlier research on the Daly River, diurnal surveys in the early dry-season (May and June) failed to detect any impact of toad invasion on crocodile numbers [42] whereas nocturnal (spotlight) surveys conducted later in the dry season (July–September) revealed a rapid 64% decline [31]. That discrepancy likely resulted from disproportionate mortality of smaller individuals, a cohort either not active, or less visible to observers during daylight hours [34].

We did not track the bait engagement, learning history and subsequent survival of individual crocodiles, because there was no way of marking individuals such that they could be identified from cameras. Crocodiles are generally marked via tail-scute clipping in mark-recapture studies, and radio telemetry was logistically impossible given the scale of our study in this rugged habitat. Instead, our conclusions rely on declining rates of bait uptake over the trial period, evidence that crocodiles stopped eating wild cane toads, the dramatic decrease in crocodile mortality following baiting in already-invaded areas (Danggu Geikie Gorge National Park), and the lack of any mass mortality events (i.e. numerous animals dying within a short period of time) in areas where toads were arriving (Bandilngan and Scrooten). These patterns suggest that crocodiles generalized their aversions from baits (fresh toad carcasses) to live toads, but we did not test that hypothesis directly. Because our taste aversion agent is salty, injecting it into baits might confound the development of taste aversion such that the crocodiles developed aversion to the baits but not to live cane toads. To test this, we could have included a third, untreated cane toad bait beside our control chicken and treated cane toad baits in our choice experiments on days 1 and 5 of trials. However, the ability of animals to detect treated and untreated food depends on their sensory capability and the primary mode of food detection [10]. Given their aquatic ambush strategies (and fast consumption of prey, often intact), and the fact that these crocodiles inhabit seasonally brackish water, taste is unlikely to be the major cue for food detection and thus memory of a food engagement or learning experience. We honed baiting methodology prior to this trial [35] to ensure a realistic context of encounter (on the edge of the water at night), prey movement (slightly moving to capture crocodiles' attention and trigger an attack), smell and taste (fresh cane toad carcasses), further cementing the predators’ learning experience. Lastly, we also needed to develop a baiting methodology that could feasibly be rolled out in the field, with large wild animals. Lithium chloride is the standard chemical used for eliciting aversion in reptiles [10], but injection or drenching of animals (as opposed to injecting LiCl into the bait) was not possible in this field study. This compromise highlights the need for *in situ* taste aversion strategies to be flexible, based on the ecology and physiology of the target species as well as limitations imposed by the conservation issue at hand. Ultimately our results demonstrated that the crocodiles developed aversions from encounters with baits, and that they then generalized this aversion to live wild toads.

Although replication at the level of sites was low in our study, our results suggest that connectivity of crocodile populations affects the impact of bait deployment. Notably, the rate of offtake of baits declined more rapidly and aversion was sustained over a longer period in isolated pools (i.e. ‘closed’ populations) than in areas contiguous with large expanses of habitat that were not baited (i.e. ‘open’ populations). Admixture of crocodiles in the latter situation may have resulted in ‘new’ individuals (naive to baits) arriving during the course of our trials, maintaining high rates of bait offtake. By contrast, most or all of the crocodiles in ‘closed’ populations rapidly learnt taste aversion. One implication of that result for management is that a lack of decline in bait consumption through time does not indicate a lack of aversion training; instead, it may mean that the baits are being taken by crocodiles from a larger area. Hence, baiting should continue even if offtake rates remain high.

In Bandilngan (Windjana Gorge) National Park, where we repeated our bait deployments in two successive years, rates of bait offtake were initially high in the second year but then fell rapidly. Given long-term philopatry of adult freshwater crocodiles [23], many of the individuals taking baits in the second year were likely the same individuals that had been trained the previous year. Presumably, some of those individuals had lost their aversion and needed a second experience to re-establish it. Although taste-aversion training is increasingly being employed in conservation programmes, few studies have quantified factors that influence the rate of learning acquisition or the rate of learning extinction [10] (but see [43]). From our observations, we suggest that managers should maintain taste-aversion training for several years after the arrival of cane toads. Even if older crocodiles eventually retain taste-aversion from one year to the next, younger individuals (the ones most at risk) may benefit from continued deployment of baits. In the longer term, as cane toads begin to breed at such sites, high numbers of small (and thus relatively non-toxic) toads in the population may facilitate aversion learning by predators without the need for continued intervention by managers [21].

A third management implication involves the timing of bait deployment. It is easier and more cost-effective to deploy baits during the late dry season, when crocodiles are concentrated in isolated pools and general access is easier. Also, shrinking water may encourage crocodiles to forage more extensively in terrestrial riparian zones, increasing rates of encounter with cane toads and the probability of fatal poisoning [25]. On the other hand, waiting too long as pools shrink may result in very high densities of crocodiles in each pool such that large territorial individuals prevent smaller animals from accessing baits. The optimal timing of deployment differs among years because annual variation in the amount of wet-season rainfall affects how soon rivers cease to flow and begin to form isolated pools [30] (Bunuba Rangers 2023, personal communication). Baiting should be timed to a period when pools are isolated but still extensive, on the cusp of the time when a scarcity of food resources and water brings crocodiles and toads into close contact (typically around September–November).

In summary, our study suggests that a relatively simple management intervention (deployment of taste-aversion-inducing baits) can buffer populations of free-living crocodiles from catastrophic decline due to invasive cane toads. In our study system with a novel toxic species, taste aversion training is best applied immediately prior to the arrival of the invasion, with the aim of curtailing the inevitable wave of predator mortality as the toads move through a new area. If let unmitigated, rapid population declines in vulnerable taxa can occur in a short period, leaving little opportunity for population recovery. However, due to the nomadic riverine ecology of freshwater crocodiles, their changes in dry-season refuge locations from year to year, and the timing of cane toad impact (around dry-season refuge pools), we show that baiting can curtail mass mortality events in the short to medium term, with reapplication extending that protection even years after toads arrive in an area.

In recent work we have documented similar success in conserving populations of a large terrestrial predator in the same ecosystems, the yellow-spotted monitor *Varanus panoptes* [21]. Methods of deployment necessarily differed considerably, with monitors trained by releasing live toad metamorphs, eggs or larvae rather than toad carcasses laced with LiCl. Nonetheless, the broad intent of the intervention—to save apex predators by inducing taste aversion—was the same. The success of these programmes suggests that buffering predator behaviour via learning may offer a useful tool in situations in which we cannot eradicate the threatening process.

## Data Availability

The supporting dataset for this research has been deposited in Dryad Digital Repository [44]. Electronic supplementary material is available from Figshare [45].

## References

[1] Beschta R, Ripple WJ. 2016 Riparian vegetation recovery in Yellowstone: the first two decades after wolf reintroduction. Biol. Conserv. **198**, 93-103. (10.1016/j.biocon.2016.03.031)

[2] Ripple WJ et al. 2014 Status and ecological effects of the world's largest carnivores. Science **343**, 1241484. (10.1126/science.1241484) 24408439

[3] Somaweera R et al. 2020 The ecological importance of crocodylians: towards evidence-based justification for their conservation. Biol. Rev. **95**, 936-959. (10.1111/brv.12594) 32154985

[4] Redpath SM, Gutiérrez RJ, Wood KA, Young JC. 2015 Conflicts in conservation: navigating towards solutions. Cambridge, UK: Cambridge University Press.

[5] Frank B, Glikman JA, Marchini S. 2019 Human–wildlife interactions: turning conflict into coexistence. Cambridge, UK: Cambridge University Press.

[6] Green SJ, Grosholz ED. 2021 Functional eradication as a framework for invasive species control. Front. Ecol. Environ. **19**, 98-107. (10.1002/fee.2277)

[7] García-Rodríguez A, Zumbado-Ulate H. 2023 Chytrid invasion drives frog redistributions. Nat. Ecol. Evol. **7**, 1587-1588. (10.1038/s41559-023-02184-9) 37567920

[8] Shine R. 2010 The ecological impact of invasive cane toads (*Bufo marinus*) in Australia. Q. Rev. Biol. **85**, 253-291. (10.1086/655116) 20919631

[9] Pettit L, Crowther MS, Ward-Fear G, Shine R. 2021 Divergent long-term impacts of lethally toxic cane toads (*Rhinella marina*) on two species of apex predators (monitor lizards, *Varanus* spp.). PLoS ONE **16**, e0254032. (10.1371/journal.pone.0254032) 34292946 PMC8297793

[10] Snijders L, Thierij NM, Appleby R, St. Clair CC, Tobajas J. 2021 Conditioned taste aversion as a tool for mitigating humanwildlife conflicts. Front. Conserv. Sci. **2**, 744704. (10.3389/fcosc.2021.744704)

[11] Tobajas J, Descalzo E, Mateo R, Ferreras P. 2020 Reducing nest predation of ground-nesting birds through conditioned food aversion. Biol. Conserv., **242**, 108405.

[12] Ternent MA, Garshelis DL. 1999 Taste-aversion conditioning to reduce nuisance activity by black bears in a Minnesota military reservation. Wildl. Soc. Bull. **27**, 720-728.

[13] Pebsworth P, Radhakrishna S. 2020 Using conditioned taste aversion to reduce human-non-human primate conflict: a comparison of four potentially illness-inducing drugs. Appl. Anim. Behav. Sci. **225**, 104948. (10.1016/j.applanim.2020.104948)

[14] Gustavson CR, Garcia J, Hankins WG, Rusiniak KW. 1974 Coyote predation control by aversive conditioning. Science **184**, 581-583. (10.1126/science.184.4136.581) 17755036

[15] Smith ME, Linnell JDC, Odden J, Swenson JE. 2000 Review of methods to reduce livestock depredation. II. Aversive conditioning, deterrents and repellents. Acta Agric. Scandinavica A **50**, 304-315. (10.1080/090647000750069502)

[16] O'Donnell S, Webb JK, Shine R. 2010 Conditioned taste aversion enhances the survival of an endangered predator imperilled by a toxic invader. J. Appl. Ecol. **47**, 558-565. (10.1111/j.1365-2664.2010.01802.x)

[17] Ward-Fear G, Pearson DJ, Brown GP, Rangers B, Shine R. 2016 Ecological immunization: *in situ* training of free-ranging predatory lizards reduces their vulnerability to invasive toxic prey. Biol. Lett. **12**, 20150863. (10.1098/rsbl.2015.0863) 26740565 PMC4785923

[18] Webb JK, Brown GP, Child T, Greenlees MJ, Phillips BL, Shine R. 2008 A native dasyurid predator (common planigale, *Planigale maculata*) rapidly learns to avoid toxic cane toads. Austral Ecol. **33**, 821-829. (10.1111/j.1442-9993.2008.01847.x)

[19] Price-Rees S, Webb J, Shine R. 2013 Reducing the impact of a toxic invader by inducing taste aversion in an imperilled native reptile predator. Anim. Conserv. **16**, 386-394. (10.1111/acv.12004)

[20] Somaweera R, Webb JK, Brown GP, Shine R. 2011 Hatchling Australian freshwater crocodiles rapidly learn to avoid toxic invasive cane toads. Behaviour **148**, 501-517. (10.1163/000579511X565763)

[21] Ward-Fear G, Rangers B, Bruny M, Everitt C, Shine R. 2024 Teacher toads: buffering apex predators from toxic invaders in a remote tropical landscape. Conserv. Lett. **17**, e13012 (10.1111/conl.13012)

[22] Bureau of Meteorology. 2023 Climate statistics for Australian locations. Canberra, Australia: Australian Bureau of Meteorology.

[23] Webb G, Manolis SC. 1989 Crocodiles of Australia. Sydney, Australia: Reed Books.

[24] Webb GJW, Manolis SC, Buckworth R. 1982 *Crocodylus johnstoni* in the McKinlay River Area, N.T. I. Variation in the diet, and a new method of assessing the relative importance of prey. Aust. J. Zool. **30**, 877-899. (10.1071/ZO9820877)

[25] Aiyer A, Shine R, Somaweera R, Bell T, Ward-Fear G. 2022 Shifts in the foraging tactics of crocodiles following invasion by toxic prey. Sci. Rep. **12**, 1267. (10.1038/s41598-021-03629-6) 35075144 PMC8786828

[26] Lever C. 2001 The cane toad: the history and ecology of a successful colonist. Otley, UK: Westbury Academic & Scientific Publishing.

[27] Shine R, Ward-Fear G, Brown GP. 2020 A famous failure: why were cane toads an ineffective biocontrol in Australia? Conserv. Sci. Pract. **2**, e296. (10.1111/csp2.296)

[28] White AW. 2003 Herpetofauna of Boodjamulla National Park and the Riversleigh World Heritage Area, north-western Queensland. Herpetofauna **33**, 65-77.

[29] Letnic M, Webb JK, Shine R. 2008 Invasive cane toads (*Bufo marinus*) cause mass mortality of freshwater crocodiles (*Crocodylus johnstoni*) in tropical Australia. Biol. Conserv. **141**, 1773-1782. (10.1016/j.biocon.2008.04.031)

[30] Britton ARC, Britton EK, McMahon CR. 2013 Impact of a toxic invasive species on freshwater crocodile (*Crocodylus johnstoni*) populations in upstream escarpments. Wildl. Res. **40**, 312-317.

[31] Fukuda Y, Tingley R, Crase B, Webb GJW, Saalfeld K. 2016 Long-term monitoring reveals declines in an endemic predator following invasion by an exotic prey species. Anim. Conserv. **19**, 75-87. (10.1111/acv.12218)

[32] Catling PC, Hertog A, Burt RJ, Forrester RI, Wombey JC. 1999 The short-term effect of cane toads (*Bufo marinus*) on native fauna in the Gulf Country of the Northern Territory. Wildl. Res. **26**, 161-185. (10.1071/WR98025)

[33] Somaweera R, Shine R. 2012 The (non) impact of invasive cane toads on freshwater crocodiles at Lake Argyle in tropical Australia. Anim. Conserv. **15**, 152-163. (10.1111/j.1469-1795.2011.00500.x)

[34] Fukuda Y, Saalfeld W, Webb G, Manolis C, Risk R. 2012 Standardised method of spotlight surveys for crocodiles in the tidal rivers of the Northern Territory, Australia. NT Nat. **24**, 14-32.

[35] Aiyer A, Rangers B, Bell T, Shine R, Somaweera R, Bruny M, Ward-Fear G. 2022 Taking the bait: Developing a bait delivery system to target free-ranging crocodiles and varanid lizards with a novel conservation strategy. Ecol. Evol. **12**, e8933. (10.1002/ece3.8933) 35784020 PMC9163195

[36] Chen W, Hudson CM, DeVore JL, Shine R. 2017 Sex and weaponry: the distribution of toxin-storage glands on the bodies of male and female cane toads (*Rhinella marina*). Ecol. Evol. **7**, 8950-8957. (10.1002/ece3.2914) 29152190 PMC5677481

[37] Paradis S, Cabanac M. 2004 Flavor aversion learning induced by lithium chloride in reptiles but not in amphibians. Behav. Processes. **67**, 11-18. (10.1016/j.beproc.2004.01.014) 15182921

[38] Letnic M, Dempster T, Jessop TS, Webb JK*.* 2024 Imperfect adaptation by freshwater crocodiles to the invasion of a toxic prey species. Biol. Invasions **26**, 1941-1955. (10.1007/s10530-024-03273-x)

[39] Tucker AD, Limpus C, McCallum H, McDonald K. 1996 Ontogenetic dietary partitioning by *Crocodylus johnstoni* during the dry season. Copeia **1996**, 978-988. (10.2307/1447661)

[40] Melotto A, Manenti R, Ficetola GF. 2020 Rapid adaptation to invasive predators overwhelms natural gradients of intraspecific variation. Nat. Commun. **11**, 3608. (10.1038/s41467-020-17406-y) 32681028 PMC7368066

[41] Pettit L, Ward-Fear G, Shine R. 2021 Invasion of cane toads (*Rhinella marina*) affects the problem-solving performance of vulnerable predators (monitor lizards, *Varanus varius*). Behav. Ecol. Sociobiol. **75**, 39. (10.1007/s00265-021-02978-6)

[42] Doody JS, Green B, Rhind D, Castellano CM, Sims R, Robinson T. 2009 Population-level declines in Australian predators caused by an invasive species. Anim. Conserv. **12**, 46-53. (10.1111/j.1469-1795.2008.00219.x)

[43] McLean LRW, Nichols MM, Taylor AH, Nelson XJ. 2022 Memory retention of conditioned aversion training in New Zealand's alpine parrot, the kea. Journ. Wild. Manag. **86**, e22221. (10.1002/jwmg.22221)

[44] Ward-Fear G. 2024 Taste aversion training can educate free-ranging crocodiles against toxic invaders [Dataset]. Dryad Digital Repository. (10.5061/dryad.qjq2bvqq5)

[45] Ward-Fear G, Bruny M, Rangers B, Forward C, Cooksey I, Shine R. 2024 Taste aversion training can educate free-ranging crocodiles against toxic invaders. *Figshare*. (10.6084/m9.figshare.c.7361696)39137886

